# Fabricating a Soft Liner-Retained Implant-Supported Palatal Lift Prosthesis for an Edentulous Patient: A Case Report

**DOI:** 10.1155/2012/203547

**Published:** 2012-06-19

**Authors:** Omid Savabi, Ebrahim Ataei, Niloufar Khodaeian

**Affiliations:** ^1^Torabinejad Dental Research Center, Department of Prosthodontics, School of Dentistry, Isfahan University of Medical Sciences, Isfahan, Iran; ^2^Department of Restorative Dentistry, School of Dentistry, Shahid Sadoughi University of Medical Sciences, Yazd, Iran; ^3^Dental Implant Research Center and Department of Prosthodontics, School of Dentistry, Isfahan University of Medical Sciences, Isfahan 81746-73461, Iran

## Abstract

This case report describes fabrication of a palatal lift prosthesis for a quadriplegic edentulous 30-year-old male with past head traumatic injury. We constructed an implant supported bar and used a soft-lining material for the maxillary palatal lift prosthesis to minimize the possibility of implant overloading and also provide a less complex and less expensive procedure for this patient.

## 1. Introduction

The possibility of survival from cerebrovascular accidents that were once fatal is increasing because of advances in medical sciences [1]. Speech disorders have been identified as a consequence of cerebrovascular accidents [1]. Paralytic dysarthria, a disorder of the nerve mechanism that controls orofacial function, often results in insufficient rhinopharyngeal closure [2].

In 1958, Gibbons and Bloomer [3] described the first successful palatal lift prostheses (PLPs) to treat paralytic dysarthria involving insufficient rhinopharyngeal closure. The purpose of PLP is to obtain rhinopharyngeal closure by displacing the soft palate to the level of normal palatal closure at the palatal plane [4]. This paper describes a technique for fabricating an implant-supported overdenture with palatal lift for an edentulous patient with past closed-head traumatic injury.

## 2. Case Report

A quadriplegic edentulous 30-year-old male with a history of head trauma caused by a car accident 3 years ago was referred for replacement of missing teeth. He had extracted all his teeth because of severe caries due to a 10-month coma and the jaw ankylosis resulting from the trauma. His chief complaint was inability of chewing, and mouth opening range was normal. After meticulous diagnostic survey, maxillary and mandibular overdentures were selected as the treatment plan. He had type 3, divisions A, D, C in different part of maxillary arch and type 1 division A in mandibular arch [5].

**Figure 1:**
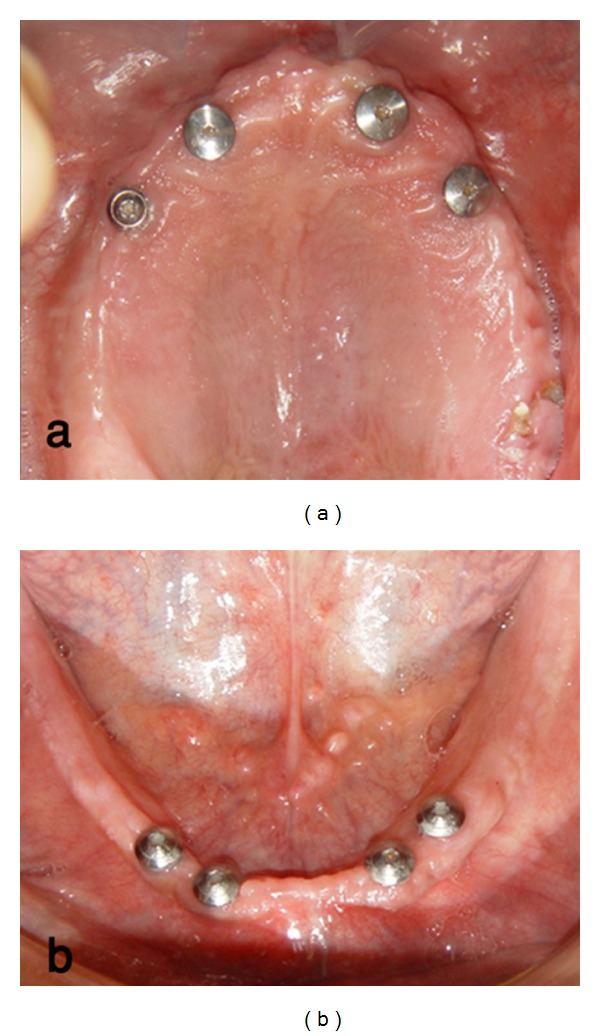
(a) Occlusal view after implant placement in maxilla. (b) Occlusal view after implant placement in mandible.

 Eight implants (4.1 × 12 mm; ITI Dental Implant System, Straumann AG, Basel, Switzerland) were inserted in the maxilla and mandible according to surgical stents (Figure 1). A bar- (ITI Dental Implant System) retained maxillary overdenture and ball- (Rhein 83, Bologna, Italy) retained mandibular overdenture were processed using conventional procedures [6].

The patient's general condition improved after a year, and his speech therapist requested palatal lift prosthesis to correct the patient's hypernasal speech and also emphasized that his soft palate muscle activities may be corrected after a period of using palatal lift prosthesis.

**Figure 2:**
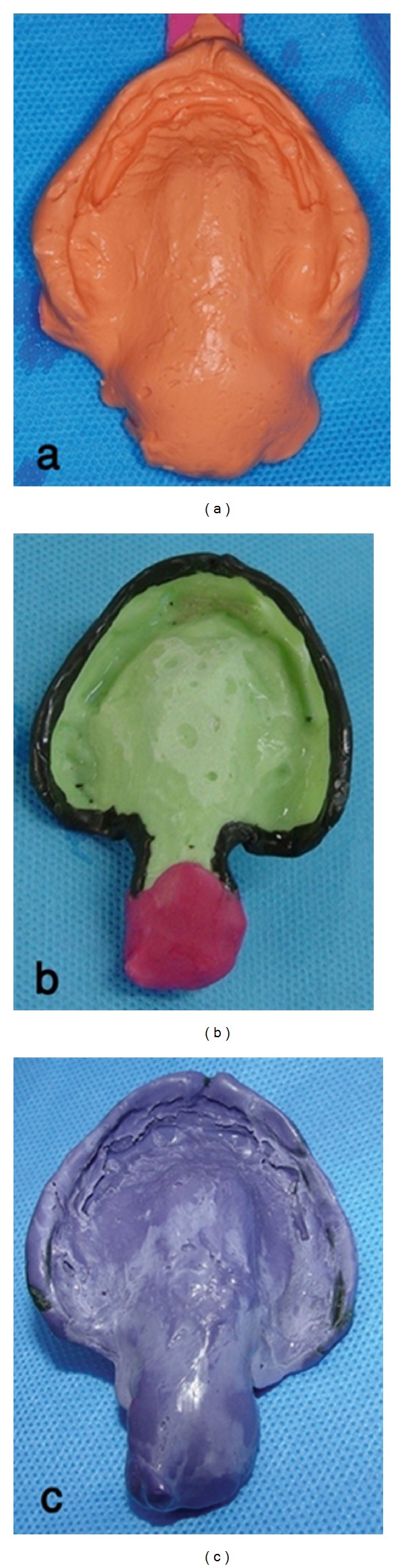
(a) Alginate impression with a prefabricated tray corrected with impression compound for extending to the soft palate site. (b) Maxillary special tray after border molding. (c) Final impression of maxillary arch extending to the soft palate.

**Figure 3:**
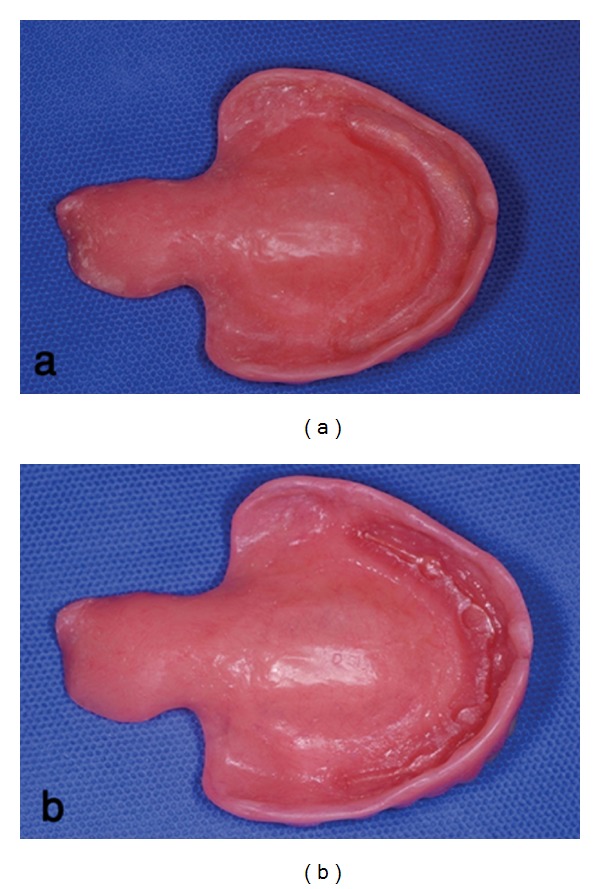
(a) Intaglio surface of the denture after spacer removal. (b) Intaglio surface of the denture after lining with resilient material.

**Figure 4:**
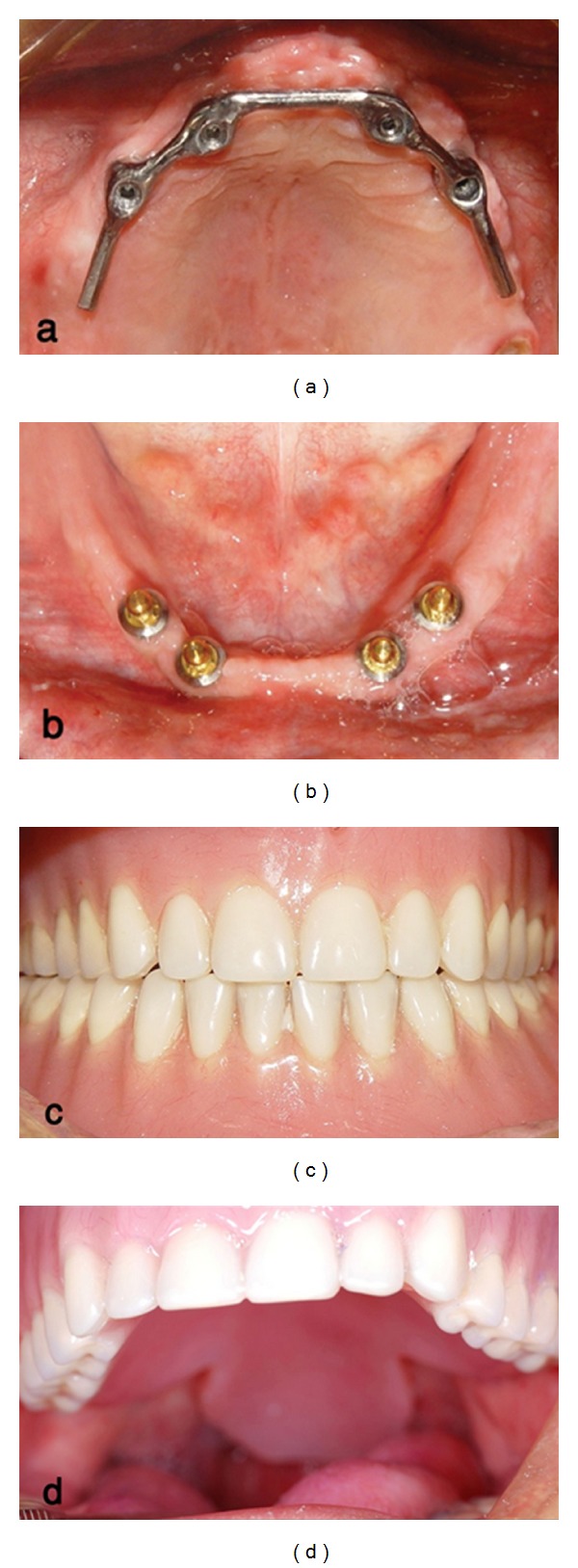
(a) Occlusal view of maxillary bar attachment. (b) Occlusal view of mandibular ball attachment. (c) Frontal view of maxillary and mandibular denture in centric occlusion. (d) Maxillary denture and palatal lift portion in place.

To construct a new maxillary prosthesis with palatal lift, preliminary impression was made with irreversible hydrocolloid (Jeltrate, Alginate, Fast set, Dentsply Philadelphia, PA, USA) and a custom tray was fabricated with light cured resin (Triad VLC, Dentsply). Border molding in vestibules was performed by impression compound (Kerr, Orange, NJ, USA) and completed with the Iso Functional compound (GC Dental Corp., Tokyo, Japan) in the soft palate site. Final impression was made by polyether material, (Impergum F, 3M ESPE, St. Paul, MN, USA, Figure 2). An overimpression of the placed mandibular overdenture with irreversible hydrocolloid was also obtained. The impressions were poured with type III stone (Fujirock, GC Dental). After obtaining the maxillomandibular records with a record base and occlusion rim, the casts were transferred to a semiadjustable articulator (ARH type, Dentatus AB, Stockholm, Sweden) using a face-bow transfer (AEB face-bow, Dentatus AB). A protrusive record was made to set the articulator's condylar elements, and a lingualized occlusal schema was achieved. Denture teeth were arranged on the record base with an index to duplicate the same position of the first overdenture teeth. The trial arrangement was evaluated intraorally, for esthetics, occlusal vertical dimension, and centric relation. The 1 mm thickness vacuum heat-pressed polyethylene was adapted around the bar portion of maxillary cast as a spacer, and the prosthesis was processed using heat cure acrylic resin material (Triplex hot, Ivoclar Vivadent, Schaan, Liechtenstein). The 1 mm thick spacer was removed from the intaglio surface of the denture, and this area was lined with autopolymerizing resilient material (Mollosil, Detax, GmbH, Ettlingen, Germany). The denture was placed on the cast until the resilient material setting was completed (Figure 3). After delivery, denture care instructions were given to the patient. Patient was told to clean the tissue surface using soft cloth. Recall appointments were scheduled at 1 day, 1 week, 1 month, and every 6 months. The soft liner replacement was scheduled every 6 months, but the soft liner maintained its resiliency after a year, so it was replaced only once in 2 years. In this period the dentures were well maintained, and the patient was comfortable using them (Figure 4). His hypernasal speech was improved, and the patient returned to use his old denture.

## 3. Discussion

In 1961, Chase introduced the use of elastic impression material to relieve traumatic tissues [7]. Resilient liners, such as Molloplast (Detax, GmbH), are widely used as a cushion on the fitting surface of dentures in the management of traumatized oral mucosa, bony undercuts, patients with bruxism parafunctions, and ridge atrophy, as well as for congenital oral defects requiring obturation [8]. Soft lining materials provide even distribution of the functional load and avoid local concentrations of stress [8–12]. There are two main types of these materials: plasticized acrylics and silicone elastomers, differing in the percentage of cross-linking agents, catalysts, and fillers which are available in autopolymerizing and heat polymerizing forms [13].

Using a soft liner-retained implant-supported overdenture offers the restorative dentist a treatment option when the number, location, or angulation of dental implants placed may differ from the original treatment plan [14]. The presence of the soft lining material compensates for the volumetric contraction of the acrylic resin that occurs during processing [15]. This prevents the dental implant components from coming into contact with the acrylic resin and minimizes the possibility of overloading implants [14].

Adding palatal lift portion to maxillary overdenture increased the off-axis load to implants, so presence of soft lining material provides an even distribution of functional load and minimizes the possibility of implant overloading. Also, construction of implant-supported bar for retention and support distributes forces among the implants [16]. It is also a less complex and less expensive procedure. In addition, because the patient suffering from a head traumatic injury may demonstrate improved soft palate function with time [4], which makes the conventional overdenture treatment options possible, fabricating a less expensive prosthesis is preferred.

A set of parameters for successful unsplinted implant-retained maxillary overdenture treatment have been published [17] including denture flanges, light retention retainer, lingualized occlusal schema, maximum implant position spread, and a minimum of 4 parallel, rough-surfaced, long, wide implants placed in denser-type bone site. Also, to minimize the effect of off-axis load splinted implants were used in this case.
